# Evaluation of the anthropometric profile and cardiometabolic results of women followed in an obesity program before and after bariatric surgery

**DOI:** 10.20945/2359-4292-2025-0041

**Published:** 2025-10-23

**Authors:** Letícia Bretones de Araujo Cavalcante, Nelson Rassi, Daniela Pultrini Pereira de Oliveira Viggiano, Adriana Ganam Alves Campos, Thiago de Souza Veiga Jardim

**Affiliations:** 1 Hospital Estadual Dr. Alberto Rassi, Goiânia, GO, Brasil; 2 Divisão de Endocrinologia, Hospital Estadual Dr. Alberto Rassi, Goiânia, GO, Brasil; 3 Programa de Pós-graduação em Ciências da Saúde, Faculdade de Medicina, Universidade Federal de Goiás, Goiânia, GO, Brasil

**Keywords:** Women, bariatric surgery, menopause

## Abstract

**Objective:**

This study assessed the effects of bariatric surgery on the anthropometric profile and cardiometabolic outcomes of women aged 28-66. Additionally, it compared data from patients under and over 50 years old who underwent bariatric surgery at a reference hospital between 2010 and 2018.

**Materials and methods:**

A retrospective cohort study analyzed the medical records of female patients aged 28-66 years at a weight control program who underwent bariatric surgery at a reference hospital over a period of 8 years. Patient profiles were characterized, normality was tested (Shapiro-Wilk), and comparisons were made between preoperative and follow-up periods (Friedman’s ANOVA test). Age groups were also compared (Mann-Whitney test). The significance level was set at 5% (p < 0.05).

**Results:**

Patients under 50 years of age had significantly greater weight loss than those over 50 years (p=0.017). However, there was no significant difference in the loss of excess weight, BMI, blood pressure, or laboratory parameters between the two groups.

**Conclusion:**

Our results reinforced the consensus that bariatric surgery is an effective treatment for overweight individuals, improving weight loss and metabolic health. Although the hormonal changes of menopause contribute to the development of an unfavorable cardiometabolic profile, bariatric surgery was equally effective in menopausal women as in younger patients in the population studied.

## INTRODUCTION

Obesity is a chronic disease influenced by nutritional, genetic, cultural, psychosocial, and behavioral factors. It is associated with various health complications, including type 2 diabetes mellitus (T2DM), hypertension (HTN), cancer, and cardiovascular diseases, leading to a reduced life expectancy (^1^,^2^). As a major global health issue, obesity contributes to millions of deaths annually. Treatment requires a multidisciplinary approach, focusing on lifestyle modifications such as diet and exercise. Although drug therapy often plays a role in supporting dietary adherence, it is seldom prescribed, particularly for older adults, due to concerns about adverse effects. Nevertheless, untreated obesity heightens the risk of complications (^3^-^8^).

Bariatric surgery is recommended for patients with class III or II obesity accompanied by comorbidities, who have not achieved success with clinical treatment over two years. This surgery is more effective for weight loss and has lower rates of recurrence, though outcomes can vary depending on the patient and the specific procedure. A study comparing sleeve gastrectomy (SG) and Roux-en-Y gastric bypass (RYGB) reported higher rates of weight regain in SG patients over five years of follow-up (^9^). Approximately one in six patients experiences a weight regain of 10% or more following bariatric surgery (^10^).

The most common procedures, RYGB and SG, constitute 90% of bariatric surgeries worldwide (^11^). According to a survey by the Brazilian Society of Bariatric and Metabolic Surgery, the number of bariatric surgeries surged by 84.73% from 2011 to 2018 (^12^). However, the total number of surgeries performed in 2022 accounted for only 1.5% of the obese population eligible for the procedure in Brazil. Despite no significant difference in obesity prevalence between genders, severe obesity, which is closely linked to comorbidities, is more common in women, as reported by the National Center for Health Statistics (^13^).

The postmenopausal state contributes to increases in total body weight. During perimenopause, elevated levels of follicle-stimulating hormone and decreased levels of estradiol and circulating sex hormone-binding globulin occur, while androgen levels are maintained, leading to relative hyperandrogenemia. These hormonal alterations modify fat distribution, resulting in greater accumulation of adipose tissue in the abdominal region and total visceral adipose tissue, thus characterizing an android distribution of fat (^14^), which increases cardiovascular risk.

Bariatric surgery has proven effective in postmenopausal women, resulting in a loss of 60%-70% of excess body weight within 12-24 months following RYGB (^15^-^17^). However, the impact of menopause on bariatric surgery outcomes remains underexplored. Asarian and cols. (^18^) indicate that reproductive axis function may influence eating behavior and weight gain. Their study on an animal model revealed that estradiol treatment in ovariectomized rats enhanced weight loss induced by gastric bypass (^18^). Ochner and cols. (^19^) observed that the reproductive axis might affect post-bariatric outcomes, with less weight loss noted in women aged 55-65 years, particularly those undergoing solely restrictive procedures, such as adjustable gastric banding (^19^).

If age-related differences in excess weight loss exist when patients undergo bariatric surgery, these differences could affect the improvement and/or resolution of obesity-related diseases.

This study aimed to evaluate the anthropometric profile and cardiometabolic outcomes of women participating in the Obesity Prevention and Control Program at Hospital Geral de Goiânia Alberto Rassi (HGG), both before and after bariatric surgery. The results were analyzed in two subgroups: women under 50 years of age, who are mostly of childbearing age, and those aged 50 and older, who are mostly in climacteric or menopause.

## MATERIALS AND METHODS

The study was approved by the Human Research Ethics Committee of the HGG (CAAE no. 43032121.3.0000.0035) and conducted in accordance with Resolution no. 466/2012 of the National Health Council. Patients’ participation was conditional upon signing an informed consent form after receiving detailed information about the research and its objectives.

This retrospective cohort study analyzed the anthropometric profiles and cardiometabolic outcomes of 106 women aged 28-66 years who underwent bariatric surgery at HGG between January 2010 and June 2018. Data were collected from medical records and analyzed statistically.

Patients whose medical records contained complete data on the presence or absence of comorbidities, medications, anthropometry, and laboratory tests from the preoperative period and at 6-12 months and 18-24 months postoperatively were included. Patients who did not meet the inclusion criteria, were lost to follow-up, died, and/or had incomplete medical records were excluded.

Body mass index (BMI), weight, excess weight loss, and the presence of comorbidities were evaluated before surgery and at postoperative time points. The use of medications for T2DM, HTN, and hypercholesterolemia was also analyzed. Results were compared between women under 50 and over 50 years. Weight change was calculated using the following formulas: percent excess body weight loss (%EBWL = [preoperative weight - current weight] x 100 / [preoperative weight - ideal weight]), percentage total weight loss (%TWL = [preoperative weight - current weight] x 100 / preoperative weight), and weight regain rate (WR = [current weight - minimum weight] x 100 / [preoperative weight - minimum weight]).

Patients with T2DM, HTN, and hypercholeste-rolemia were evaluated for remission, improvement, recurrence, or lack of improvement post-surgery. Remission was defined as the improvement of blood glucose (^20^), blood pressure, or cholesterol levels without medication. Improvement was indicated by a decrease in medication dosage or the number of classes. Recurrence was defined as the reintroduction of medication after remission, and no improvement was indicated by the continued use of the same medication.

The patient profile was characterized using absolute frequency, mean, standard deviation, median, and interquartile range. Normality was verified using the Shapiro-Wilk test. Variation (delta) between age groups was compared using the Mann-Whitney test, with a significance level of 5% (α = 0.05).

## RESULTS

### Preoperative anthropometric and cardiometabolic profiles of patients

Between January 2010 and June 2018, 493 patients underwent bariatric surgery at HGG: 84 (17%) were men and 409 (83%) were women. The number of surgical procedures increased significantly over the years, especially in 2018, which accounted for nearly half of all procedures during the study period. Out of the 409 women, medical records for 106 patients were selected primarily due to loss of follow-up and incomplete data. All patients (100%) underwent RYGB surgery. The average age of the patients before surgery was 44.1 years, ranging from 28 to 66 years. Most patients (70.8%) were between 28 and 50 years old. The average weight and BMI at baseline were 122.78 kg and 47.93 kg/m^2^, respectively. The majority (93.3%) had class III obesity. The most prevalent comorbidity was HTN (67.9%), followed by hypercholesterolemia (65.1%) and T2DM (46.2%).

Patients aged 50 years or older had a higher prevalence of HTN and dyslipidemia compared to those younger than 50 years. There was no significant difference in BMI between these two groups (*p* = 0.143) (**Table 1**). None of the patients over 50 used menopausal hormone therapy at any evaluated time.

**Table 1:** Patients’ profiles in the preoperative period according to age group (n = 106)

Variables	Age range (years)			Total	P
	< 50 (n = 75)	≥ 50 (n = 31)		n = 106	
Age (years)^*^	39.27 ± 5.66	58.19 ± 4.66		44.80 ± 10.18	**<0.001**
Height (m)^*^	1.61 ± 0.07	1.57 ± 0.06		1.60 ± 0.07	**0.002**
Weight (kg)^*^	126.31 ± 20.23	114.24 ± 14.61		122.78 ± 19.49	**0.003**
BMI (kg/m^2^)^*^	48.53 ± 7.17	46.46 ± 4.76		47.93 ± 6.60	0.143
OADs^**^	1.00 (1.00-2.00)	1.00 (1.00-2.00)		1.00 (1.00-2.00)	0.062
Insulin^**^ (UI/kg/day)	0.65 (0.52-0.74)	0.47 (0.29-0.62)		0.605 (0.40-0.66)	0.282
AntiHTN^**^	2.00 (2.00-3.00)	2.00 (2.00-3.00)		2.00 (2.00-3.00)	**0.037**
Hypolipemiant^**^	1.00 (1.00-1.00)	1.00 (1.00-1.00)		1.00 (1.00- -1.00)	**<0.001**
T2DM n (%)					
No	44 (58.7%)	13 (41.9%)		57 (53.8%)	0.116
Yes	31 (41.3%)	18 (58.1%)		49 (46.2%)	
HTN n (%)					
No	30 (40.0%)	4 (12.9%)		34 (32.1%)	**0.007**
Yes	45 (60.0%)	27 (87.1%)		72 (67.9%)	
DLD n (%)					
No	35 (46.7%)	2 (6.5%)		37 (34.9%)	**<0.001**
Yes	40 (53.3%)	29 (93.5%)		59 (65.1%)	

* Shapiro-Wilk test;

** Median and interquartile range (P25-75).

n, absolute frequency; %, relative frequency.

AntiHTN: antihypertensive drugs; BMI: body mass index; DLD: dyslipidemia; HTN: hypertension; OADs: oral antidiabetic drugs; T2DM: type 2 diabetes mellitus.

### Postoperative anthropometric and cardiometabolic profiles of patients

The postoperative anthropometric and cardiome-tabolic profiles of patients were examined. This sample of patients (n = 106), who had a mean weight of 122.78 kg (±19.49 kg) before surgery, experienced a mean weight loss of 42.44 kg (±14.15 kg). Consequently, the mean BMI decreased from 47.93 (±6.60 kg/m^2^) to 31.43 kg/m^2^ (±5.27 kg/m^2^) at 18-24 months postoperatively. The mean excess weight loss was 74.70% (±17.59%), and the mean weight regain rate was 1.87% (±4.93%) during the 18-24 PO months.

Considering remission rates, T2DM had the highest resolution after 18-24 months of bariatric surgery, at a rate of 81.7%. Remission of dyslipidemia and HTN occurred in 78.3 and 66.7% of patients, respectively. Hypertension and dyslipidemia were associated with higher no-improvement rates (13.9 and 14.5%, respectively) compared to T2DM (4.1%).

### Comparative analysis between women under 50 years old and those aged ≥ 50 years old: anthropometric data and comorbidities at 18-24 months PO follow-up

Patients younger than 50 years, compared to those aged ≥ 50 years, did not show a significant difference in mean excess weight loss (76.35 ± 16.62% *vs.* 70.70 ± 19.44%, respectively; *p* = 0.13) or in the percentage of weight loss (35.08 ± 7.96% *vs.* 31.88 ± 9.07%, respectively; *p* = 0.118).

There was no difference between the two groups regarding changes (∆) in BMI, blood pressure, or the following laboratory parameters: glucose, HbA1c, total cholesterol, and lipoprotein fractions (**Table 2**).

**Table 2:** Preoperative vs. 18-24 months delta comparison of patients aged < 50 and ≥ 50 years (n = 106)

Variables	Age group (∆)		p^*^
	< 50 years (P25-75)	≥ 50 years (P25-75)	
BMI (kg/m^2^)	-16.37 (-19.29 - -13.48)	-14.95 (-18.06 - -12.22)	0.087
SBP (mmHg)	-5.00 (-17.50 - 0.00)	-10.00 (-20.00 - 0.00)	0.385
DBP (mmHg)	0.00 (-10.00 - 0.00)	-10.00 (-10.00 - 0.00)	0.954
TC (mg/dL)	-21.00 (-47.50 - 1.50)	-25.00 (-53.50 - -2.50)	0.649
HDL-c (mg/dL)	10.00 (2.00 - 22.00)	9.00 (0.00 - 15.50)	0.773
LDL-c (mg/dL)	-17.00 (-38.00 - -1.00)	-22.00 (-52.00 - -10.00)	0.726
TG (mg/dL)	-55.00 (-107.00- -25.00)	-39.00 (-84.00 - -18.00)	0.283
FPG (mg/dL)	-17.00 (-31.50 - -8.00)	-16.00 (-24.00 - -4.50)	0.381
HbA1c (%)	-1.05 (-2.05 - -0.27)	-0.70 (-2.65 - -0.30)	0.966
%EBWL (%)	76.35 ± 16.62	70.70 ± 19.44	0.130
Weight regain (%)	1.81 ± 4.28	2.02 ± 6.31	0.834

BMI: body mass index; DBP: diastolic blood pressure; FPG: fasting plasma glucose; HbA1c: glycated haemoglobin; HDL-c: high-density lipoprotein cholesterol; LDL-c: low-density lipoprotein cholesterol; SBP: systolic blood pressure; TC: total cholesterol; TG: tryglicerides; %EBWL: percent excess body weight loss.

* Mann-Whitney test.

## DISCUSSION

Obesity in Brazil is on the rise, with projections indicating that it could affect nearly 30% of adults by 2030 (^21^), posing a significant risk for cardiovascular disease, the leading cause of death globally, claiming approximately 18 million lives annually. The subs-tantial and sustained weight reduction following ba-riatric surgery is linked to significant decreases in cardiovascular risk factors such as T2DM, HTN, and hypertriglyceridemia, underlining the importance of early detection and management of these conditions, as recommended by the World Health Organization (^22^).

In the study conducted by Castanha and cols. (^23^), 103 patients, both male and female, aged between 22 and 63 years underwent either GS surgery (n = 40) or RYGB (n = 63), with the majority being female (89.3%). The assessment of %EBWL and BMI before and after surgery, with a mean follow-up period of 41.87 months (±37.35), demonstrated that the mean preoperative BMI was 48.10 kg/m^2^ and the postoperative BMI was 31.05 kg/m^2^, which is consistent with our findings (47.93 and 31.43 kg/m^2^, respectively). Additionally, the mean percentage of excess weight lost reached 69.35%, which is comparable to the 74.7% observed in our study. Nonetheless, the preoperative prevalence rates of HTN, dyslipidemia, and T2DM were lower in the study by Castanha and cols. (^23^) (42.4, 9.4, and 18.2%, respectively), yet the resolution rates for HTN (70.8%), T2DM (80.7%), and dyslipidemia (68.8%) were similar to those found in the current study.

Bariatric surgery has proven effective in postmenopausal women, leading to 60%-70% excess body weight loss within 12-24 months with RYGB (^15^-^17^). Nevertheless, the influence of menopause on bariatric surgery outcomes has been under-explored. Asarian and cols. (^18^) reported enhanced weight loss following estradiol administration in oophorectomized rats undergoing gastric bypass. Ochner and cols. (^19^) observed that the weight loss effect of laparoscopic adjustable gastric banding diminished in postmenopausal women (55-65 years old) compared to women of reproductive age (20-45 years old). In that study, the excess body weight loss was greater in women aged 20-45 years than in those aged 55-65 years who underwent laparoscopic adjustable gastric banding 12 and 24 months post-surgery (*p* = 0.016), amounting to approximately 7 kg more in younger women. However, this effect was not observed in women undergoing RYGB (*p* = 0.24). Among men, no differences were found between the two surgical techniques in terms of weight loss. The article did not reference the metabolic profile impact of this smaller reduction in excess weight percentage in the group undergoing restrictive surgery.

Menopause typically occurs around 50 years of age, with 90% of cases appearing between 45-55 years old (^24^). In this study, participants were divided into two groups based on the average age of 50 years: those aged < 50 years (likely still of childbearing age) and those aged ≥ 50 years (likely in the climacteric or menopausal phase). Although data suggest a trend towards greater mean excess weight loss in patients aged < 50 years (76.35% ± 16.62) compared to those over 50 years (70.70% ± 16.62), the difference was not statistically significant (*p* = 0.13).

Differences in preoperative prevalence of comorbidities were significant between participants under 50 years and those over 50, with older patients showing higher rates of HTN and dyslipidemia. However, no differences were found between the groups regarding changes (∆) in BMI, blood pressure, or laboratory parameters when comparing preoperative and postoperative values. This suggests that bariatric surgery was equally effective in improving health outcomes across both age groups in the study population.

Walędziak and cols. (^25^) examined 614 women undergoing bariatric surgery (GS or RYGB), categorizing them by menopausal status (menopause was defined as fewer than three menstrual cycles in the year preceding the data collection in patients aged 45 years or older). The percentages of excess weight loss (87.8% vs. 73.8%; *p* < 0.01) and total weight loss (50 kg vs. 43 kg; *p* < 0.001) were lower in the postmenopausal group compared to premenopausal women, contrary to what was found in the evolution of the Obesity Prevention and Control Program patients.

This study highlighted similarities in the resolution of comorbidities post-bariatric surgery, although its scope was limited by the small number of participants meeting inclusion criteria. Data collection from medical records led to the exclusion of many patients due to incomplete information or loss to follow-up, reducing the study’s sample size to 25% of the total bariatric surgery patient population. This is a common problem that weakens retrospective analyses but mirrors real -life scenarios. The rate of noncompliance with postoperative follow-up after bariatric surgery is significant, varying with study and context. Medical literature reveals that follow-up noncompliance rates can be notably high, with one study indicating a rate of 29.7% (^26^), and another demonstrating a decrease in adherence over time, with only 6.5% of patients maintaining follow-up at 24 months (^27^). Furthermore, a national study in France showed a decline in surgeon visits from 87.1% in the first year to 29.6% in the fifth year (^28^).

Additional study limitations include the assumption of menopause without clinical or hormonal evaluation. Moreover, the study’s external validity is limited due to it being conducted at a single center, and the data exclusively covers patients who underwent RYGB.

In conclusion, remission and/or improvement of comorbidities are essential parameters in evaluating the effectiveness of bariatric surgery, as the presence of T2DM, HTN, and dyslipidemia can influence the indication for the procedure. In this study, the intervention’s effects on the analyzed variables were uniform among patients aged < 50 and ≥ 50 when comparing preoperative values with those at 18-24 months PO. Although hormonal changes associated with menopause contribute to the development of an adverse cardiometabolic profile, bariatric surgery in this age group proved to be as effective as it did in patients under 50 years of age in terms of clinical and laboratory improvements.

## Data Availability

datasets related to this article will be available upon request to the corresponding author.
